# *Kankuamo*, a new theraphosid genus from Colombia (Araneae, Mygalomorphae), with a new type of urticating setae and divergent male genitalia

**DOI:** 10.3897/zookeys.601.7704

**Published:** 2016-06-29

**Authors:** Carlos Perafán, William Galvis, Miguel Gutiérrez, Fernando Pérez-Miles

**Affiliations:** 1 Sección Entomología, Facultad de Ciencias, Universidad de La República, Iguá 4225, Montevideo, Uruguay.+57-3143188819; 2 Laboratorio de Aracnología & Miriapodología, Instituto de Ciencias Naturales, Departamento de Biología, Universidad Nacional de Colombia, Bogotá, Colombia; 3 Ecología y Biodiversidad en Ecosistemas Tropicales (EBET), Facultad de Ciencias Básicas, Universidad de La Guajira, Riohacha, Colombia

**Keywords:** New species, Sierra Nevada de Santa Marta, Theraphosinae phylogeny, urticating setae type VII

## Abstract

A new monotypic Theraphosidae genus, *Kankuamo* Perafán, Galvis & Pérez-Miles, **gen. n.**, is described from Colombia, with a new type of urticating setae. These setae differ from others principally by having a small distal oval patch of lanceolate reversed barbs. Males of *Kankuamo* **gen. n.** additionally differ by having a palpal bulb organ very divergent from all known species, with many conspicuous keels dispersed across the median tegulum to the tip, mostly with serrated edges. Females differ by having spermathecae with a single notched receptacle, with two granulated lobes and several irregular sclerotized longitudinal striations. The new urticating setae, type VII, is characterized, illustrated and its releasing mechanism is discussed. It is hypothesized that these setae are the first in Theraphosinae subfamily whose release mechanism is by direct contact. *Kankuamo* **gen. n.** is described and illustrated on the basis of the type species *Kankuamo marquezi* Perafán, Galvis & Gutiérrez, **sp. n.**, and their remarkable characteristics, morphological affinities and cladistic relationship are analyzed.

## Introduction

Theraphosidae Thorell, 1869 is the most speciose of the Mygalomorphae with more than 130 genera and 980 species (World Spider Catalogue 2015), mainly distributed in the tropical and subtropical regions, and currently divided into 11 subfamilies (Guadanucci 2014). This family comprises large sized and setose spiders commonly known as tarantulas in the New World. A unique morphological characteristic of most New World theraphosids is the presence of defensive urticating setae (Cooke et al. 1972). This defense mechanism is found in roughly 540 of the 600 theraphosid Neotropical species (Bertani and Guadanucci 2013). Representatives of all known species of the subfamily Theraphosinae, as well as species of the Aviculariinae genera *Avicularia* Lamarck, 1818, *Ephebopus* Simon, 1892, *Iridopelma* Pocock, 1901, *Pachistopelma* Pocock, 1901, and *Typhochlaena* C.L. Koch, 1850 have urticating setae. The arboreal tarantulas *Tapinauchenius* Ausserer, 1871 and *Psalmopoeus* Pocock, 1895, and several ‘Ischnocolinae’ genera are the only New World theraphosids that lack any urticating setae (Bertani and Guadanucci 2013).

The morphological characteristics of urticating setae have long been used in taxonomy and systematics of Theraphosidae, being useful as a set of characters for differentiation of subfamilies and genera as shown in phylogenetic analysis (Cooke et al. 1972, Raven 1985, Pérez-Miles et al. 1996, Pérez-Miles 2002, Perafán 2010, Bertani and Guadanucci 2013). Six different types of urticating setae have been described based on their morphology, ornamentation, length and releasing mechanism; two types are known to Aviculariinae (II and V), and another four in Theraphosinae (I, III, IV and VI) (Cooke et al. 1972, Marshall and Uetz 1990, Pérez-Miles 1998) (see Bertani and Guadanucci 2013, Figure 1). Excepting type V which occurs on the distal prolateral surface of the palpal femora (Marshall and Uetz 1990, Foelix et al. 2009), all other types are found on the dorsum of the abdomen.

**Figure 1.**
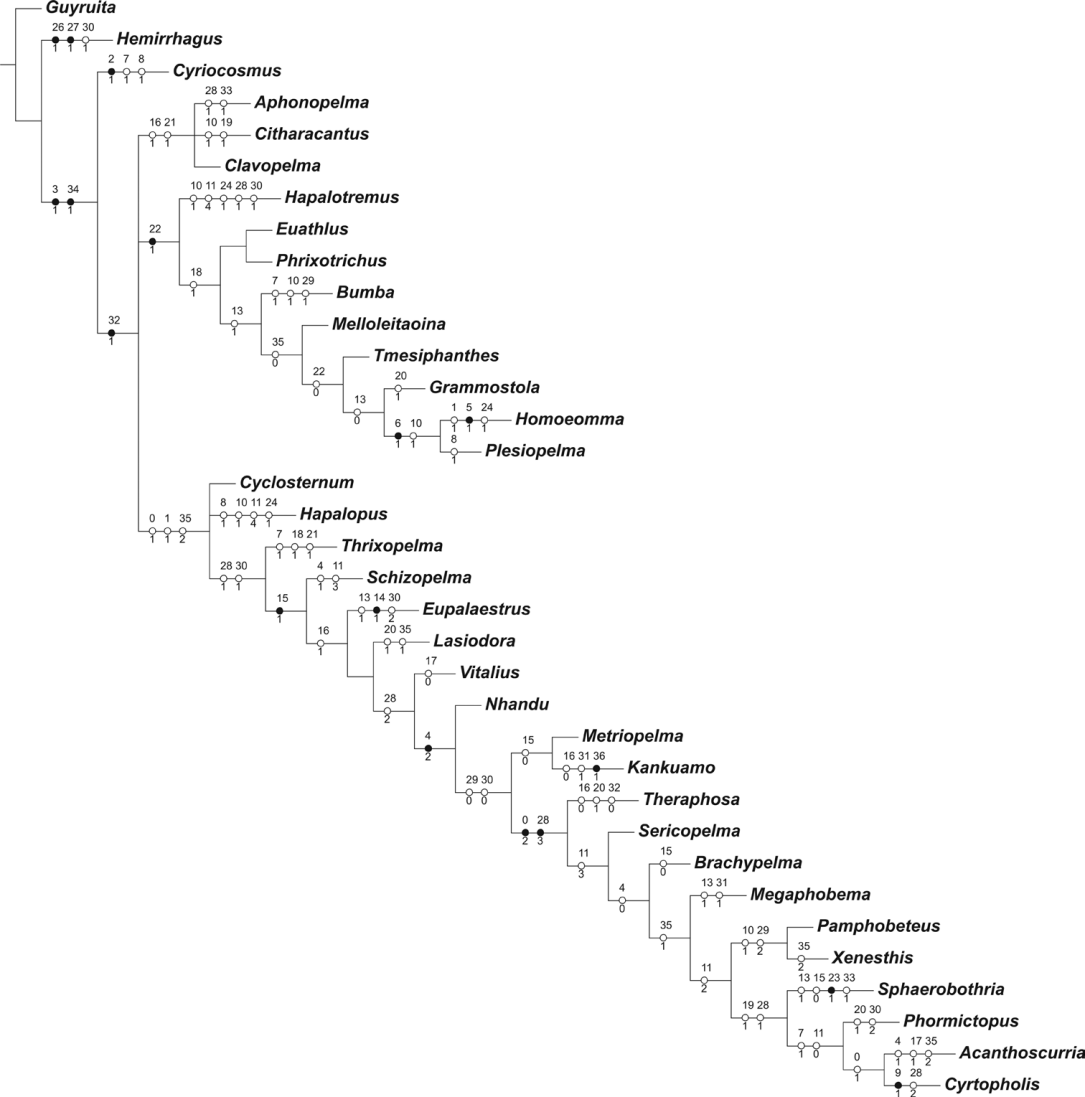
Preferred phylogeny of Theraphosinae. Strict consensus of three trees (maximum parsimony, heuristic search and implied weighting k = 8, L = 127; Ci = 36; Ri = 64). Black and white circles represent homologous and homoplastic characters, respectively.

During study of Colombian tarantulas, we discovered specimens from Sierra Nevada de Santa Marta, Colombia, with a different type of urticating setae which did not fit with any known types. These setae mainly differ by having a small distal patch of reversed lanceolate barbs (regarding the main barbs; *sensu* Cooke et al. 1972) near the penetrating tip, and small main barbs that extend along the whole seta from the apex. Additionally, males present a palpal bulb remarkably different from all known Theraphosidae, with a large number of conspicuous keels on tegulum and embolus. The morphology and arrangement of these keels do not easily fit with the palpal bulb homologies proposed by Bertani (2000) for the Theraphosinae. However, this taxon shares the main general characteristics of the Theraphosinae subfamily, extended subtegulum, keels on palpal bulb, and urticating setae (Raven 1985, Pérez-Miles et al. 1996). Females differ from other Theraphosinae genera by having spermathecae with a single notched receptacle with two granulated lobes and several irregular sclerotized longitudinal striations.

Based on its unique combination of characters, we propose the new monotypic Theraphosinae genus *Kankuamo* Perafán, Galvis & Pérez-Miles, gen. n., which is here diagnosed, described and illustrated on the basis of the type species *Kankuamo marquezi* Perafán, Galvis and Gutiérrez sp. n. Morphological aspects are discussed and its phylogenetics relationship are analyzed based on a Theraphosinae cladistic re-analysis presented in this paper. Considering the size and morphology of the urticating setae in *Kankuamo* gen. n., we propose them as a novel type, here naming them as type VII urticating setae. These setae are described and illustrated, and their releasing mechanism is discussed.

## Material and methods

Urticating setae terminology follows Cooke et al. (1972) and Bertani and Guadanucci (2013). Male palpal organ keel terminology follows Bertani (2000). Number and disposition of spines are enumerated from the anterior third to the posterior third, modified from Petrunkevitch (1925). Spination was recorded from the right-side limbs. All measurements were taken using an ocular micrometer and are given in millimeters (mm). Leg and palp measurements were taken on the dorsal side, along the central axis of the left-side limbs. The genital structures were removed for examination: either male left palpal bulb, or female spermathecae. Urticating setae from different regions of the abdomen were removed and examined under optical microscope and scanning electron microscope (SEM). Photographs were taken with an Infinity Lite camera adapted to the stereoscope lens (Nikon SMZ-10), SEM with JEOL JSM-5900 LV. The geographic coordinates are taken by GPS, Datum WGS84. The distribution map was produced using DIVA-GIS version 7.5.0 (http://www.diva-gis.org/). The type material is stored in 70% ethanol, and deposited in the Instituto de Ciencias Naturales Arachnological Collection (ICN-Ar), at the Universidad Nacional de Colombia, Bogotá, Colombia.

Abbreviations are listed below:

AcK
 accessory keels 


AK
 apical keel 


ALE
 anterior lateral eyes 


AME
 anterior median eyes 


ap
 apical 


D
 dorsal 


DKs
 dorsal keels 


ICN
 Instituto de Ciencias Naturales, Universidad Nacional de Colombia, Bogotá, Colombia 


k
 concavity constant 


OQ
 ocular quadrangle (including lateral eyes) 


P
 prolateral 


PIK
 prolateral inferior keel 


PME
 posterior median eyes 


PMS
 posterior median spinnerets 


PLE
 posterior lateral eyes 


PLS
 posterior lateral spinnerets 


PSK
 prolateral superior keel 


SAK
 subapical keel 


SpAcK
 supra-accessory keels 


R
 retrolateral 


V
 ventral 


**Cladistic analysis.** Cladistic analysis was based on the previous matrix of Theraphosinae genera used by Pérez-Miles et al. 1996 and Pérez-Miles 2000 with some modifications. This matrix was completed as far as possible, including material examined and literature, complemented with the new evidences of the palpal bulb homology proposed by Bertani (2000, 2001), and the characters used on the matrix of Perafán and Pérez-Miles (2014). The original matrix from Pérez-Miles et al. 1996 was modified on characters referred to palpal bulb keels (characters 3 and 4) and replaced with those proposed by Bertani (2000, 2001), Perafán and Pérez-Miles (2014). Character 13 related to the spermathecae shape was also amended. Furthemore, the hypotehical outgroup used on the analysis previous (Pérez-Miles et al 1996, Pérez-Miles 2000) was modified by a Schismatothelinae taxon *Guyruita* Guadanucci et al., 2007 (Guadanucci 2014) and the terminal *Pseudotheraphosa* Tinter, 1991 was eliminated because it’s a junior synonym of *Theraphosa* Thorell, 1869 (Bertani 2001).

A data matrix composed of 37 morphological characters and 35 genera has been constructed (Table 1). The cladistics analysis was carried out in TNT version 1.1 (Goloboff et al. 2008), under maximum parsimony. The characters were polarized according to the out-group criterion (Watrous and Wheeler 1981), and all characters treated unordered, with other settings as in Pérez-Miles et al. (1996). A heuristic search was used with 15 addition sequences and tree-bisection reconnection processes, with and without implied weighting (Goloboff 1993) under different concavity values (k = 3–12). Character optimization was performed in Winclada 1.00.08 (Nixon 1999-2002) and characters discussed below are those that are unambiguously optimized.

**Table 1.** Character matrix used in cladistic analysis of Theraphosinae genera. (?) inapplicable, unknown or doubtful.

	0	1	2	3	4	5	6	7	8	9	1 0	1 1	1 2	1 3	1 4	1 5	1 6	1 7	1 8	1 9	2 0	2 1	2 2	2 3	2 4	2 5	2 6	2 7	2 8	2 9	3 0	3 1	3 2	3 3	3 4	3 5	3 6
*Guyruita*	0	0	0	0	0	0	0	0	0	0	1	0	0	0	0	0	0	0	0	0	0	0	0	0	0	0	0	0	0	0	0	0	0	0	0	0	0
*Acanthoscurria*	1	1	0	1	1	0	0	1	0	0	?	0	1	0	0	1	1	1	0	1	0	0	0	0	0	0	0	0	1	0	0	0	1	0	1	2	0
*Aphonopelma*	0	0	0	1	0	0	0	0	0	0	0	0	1	0	0	0	1	0	0	0	0	1	0	0	0	0	0	0	1	0	0	0	1	1	?	1	0
*Brachypelma*	2	1	0	1	0	0	0	0	0	0	0	3	1	0	0	0	1	1	0	0	0	0	0	0	0	0	0	0	3	0	0	0	1	0	1	2	0
*Bumba*	0	0	0	1	0	0	0	1	0	0	1	0	1	1	0	0	0	1	1	0	0	0	1	0	0	0	0	0	0	1	0	0	?	0	1	1	0
*Citharacantus*	0	0	0	1	0	0	0	0	0	0	1	0	1	0	0	0	1	0	0	1	0	1	0	0	0	0	0	0	0	0	?	0	1	0	1	1	0
*Clavopelma*	0	0	0	1	0	0	0	0	0	0	0	0	1	0	0	0	1	1	0	0	0	1	0	0	0	0	0	0	?	?	?	?	?	?	?	?	0
*Cyclosternum*	1	1	0	1	0	0	0	0	0	0	0	0	1	0	0	0	0	1	0	0	0	0	0	0	0	0	0	0	0	0	0	0	1	0	1	2	0
*Cyriocosmus*	0	0	1	1	0	0	0	1	1	0	0	0	1	0	0	0	0	0	0	0	0	0	0	0	0	0	0	0	0	0	0	0	0	0	1	1	0
*Cirtopholis*	1	1	0	1	0	0	0	1	0	1	0	0	1	0	0	1	1	0	0	1	0	?	0	0	0	0	0	0	2	0	0	0	1	0	1	1	0
*Euathlus*	0	0	0	1	0	0	0	0	0	0	0	1	1	0	0	0	0	1	1	0	0	0	1	0	0	0	0	0	0	0	0	0	1	0	1	1	0
*Eupalaestrus*	1	1	0	1	0	0	0	0	0	0	0	0	1	1	1	1	1	1	0	0	0	0	0	0	0	0	0	0	1	1	2	0	1	0	1	2	0
*Grammostola*	0	0	0	1	0	0	0	0	0	0	0	0	1	0	0	0	0	1	1	0	1	0	0	0	0	0	0	0	0	0	0	0	1	0	1	0	0
*Hapalopus*	1	1	?	1	0	0	0	0	1	0	1	4	1	0	0	0	0	1	0	0	0	0	0	0	1	0	0	0	0	0	0	0	1	0	1	2	0
*Hapalotremus*	0	0	0	1	0	0	0	0	0	0	1	4	1	0	0	0	0	1	0	0	0	0	1	0	1	0	0	0	1	0	1	0	1	0	1	1	0
*Hemirrhagus*	0	0	0	0	0	0	0	0	0	0	0	0	1	0	0	0	0	0	0	0	0	0	0	0	?	0	1	1	0	0	1	0	0	0	0	1	0
*Homoeomma*	0	1	0	1	0	1	1	0	0	?	1	0	1	0	0	0	0	1	1	0	0	0	0	0	1	0	0	0	0	0	0	0	1	?	1	0	0
*Lasiodora*	1	1	0	1	0	0	0	0	0	0	0	2	1	0	0	1	1	1	0	0	1	0	0	0	0	0	0	0	1	1	1	0	1	0	1	1	0
*Megaphobema*	2	1	0	1	0	0	0	0	0	0	0	3	1	1	0	1	1	1	0	0	0	0	0	0	0	0	0	0	3	1	0	1	1	0	1	1	0
*Melloleitaoina*	0	0	0	1	0	0	0	0	0	0	0	0	1	1	0	0	0	1	1	0	0	0	1	0	0	0	0	0	0	0	0	0	1	0	1	0	0
*Metriopelma*	1	1	0	1	2	0	0	0	0	0	?	?	1	?	0	0	1	?	0	0	0	0	0	0	0	0	0	0	?	?	0	0	1	0	1	2	0
*Nhandu*	1	1	0	1	2	0	0	0	0	0	0	2	1	0	0	1	1	1	0	0	0	0	0	0	0	0	0	0	2	2	1	0	1	0	1	2	0
*Pamphobeteus*	2	1	0	1	0	0	0	0	0	0	1	2	1	0	0	1	1	1	0	0	0	0	0	0	0	0	0	0	3	2	0	0	?	0	1	1	0
*Phrixotrichus*	0	0	0	1	0	0	0	0	0	0	0	1	?	0	0	0	0	1	1	0	0	0	1	0	0	0	0	0	0	0	0	0	1	0	1	1	0
*Phormictopus*	2	1	0	1	0	0	0	1	0	0	0	0	1	0	0	1	1	0	0	1	1	0	0	0	0	0	0	0	1	0	2	0	1	0	1	1	0
*Plesiopelma*	0	0	0	1	0	0	1	0	?	0	1	0	1	0	0	0	0	1	1	0	0	0	0	0	0	0	0	0	0	0	0	0	1	0	1	0	0
*Schizopelma*	1	1	0	1	1	0	0	0	0	0	?	3	1	0	0	1	0	1	0	0	0	0	0	0	0	0	0	0	?	?	?	0	1	0	1	?	0
*Sericopelma*	2	1	0	1	2	0	0	0	0	0	?	3	1	0	0	1	1	1	0	0	0	0	0	0	0	0	0	0	3	0	0	0	?	?	1	2	0
*Sphaerobothria*	2	1	0	1	0	0	0	0	0	0	0	2	1	1	0	0	1	0	0	1	0	0	0	1	0	0	0	0	1	0	0	0	1	1	1	1	0
*Theraphosa*	2	1	0	1	2	0	0	0	0	0	?	2	1	0	0	1	0	1	0	0	1	0	0	0	0	0	0	0	3	0	0	0	0	0	1	2	0
*Thrixopelma*	1	1	0	1	0	0	0	1	0	0	0	0	1	0	0	0	0	1	1	0	0	1	0	0	0	0	0	0	1	0	1	0	1	0	1	2	0
*Tmesiphanthes*	0	0	0	1	0	0	0	0	0	0	0	0	1	1	0	0	0	1	1	0	0	0	0	0	0	0	0	0	0	0	0	0	1	0	1	0	0
*Vitalius*	1	1	0	1	0	0	0	0	0	0	0	2	1	0	0	1	1	0	0	0	0	0	0	0	0	0	0	0	2	2	1	0	1	0	1	2	0
*Xenesthis*	2	1	0	1	0	0	0	0	0	0	1	2	1	0	0	1	1	0	0	0	0	0	0	0	0	0	0	0	3	2	0	0	1	0	1	2	0
***Kankuamo***	1	1	0	1	2	0	0	0	0	0	?	2	1	0	0	0	0	0	0	0	0	0	0	0	0	0	0	0	2	0	0	1	1	0	1	2	1

**Data set.** Characters used in the cladistic analysis. The data matrix is listed in Table 1.

Pérez-Miles et al. 1996, Pérez-Miles 2000 Characters: (0) Apical region of palpal bulb: subcylindrical = 0; subconical = 1; cancave-convex = 2. (1) Relative width of sclerites II+III of bulb: norrow (less than 10% of length) = 0; wide = 1. (2) Paraembolic apophysis: absent = 0; present = 1. (3) Subtegulum: not extended = 0; large extended = 1. (4) Male tibial apophysis (leg I): double = 0; one = 1; absent = 2. (5) Digitiform apophysis of bulb: absent = 0; present = 1. (6) Metatarsus I of male: without basal process = 0; with basal process = 1. (7) Male palpal tibia: without retrolateral process = 0; with retrolateral process = 1. (8) Male palpal tibia: without retrolateral cluster of spines = 0; with retrolateral cluster of spines. (9) Male palpal tibia: without prolateral process = 0; with prolateral process = 1. (10) Flexion of metatarsus I on males: on outer side of tibial spurs = 0; between tibial spurs = 1. (11) Spermathecae (modified character): two separated longitudinal seminal receptacles = 0; two separated transversal seminal receptacles = 1; two seminal receptacles widely fused = 2; single semicircular receptacle = 3; single oval receptacle = 4. (12) Spermathecae: multilobular in each side = 0; unilobular al least in each side = 1. (13) Femur III: not incrassate = 0; incrassate = 1. (14) Tibia IV: not incrassate = 0; incrassate = 1. (15) Femur IV: without retrolateral scopula = 0; with retrolateral scopula = 1. (16) Urticating setae type I: absent = 0; present = 1. (17) Urticating setae type III: absent = 0; present = 1. (18) Urticating setae type IV: absent = 0; present = 1. (19) Trochanteral stridulatory setae: absent = 0; present = 1. (20) coxal stridulatory setae: absent = 0; present = 1. (21) Coxal spinules: absent = 0; present = 1. (22) Labial cuspules: numerous (more than 15) = 0; few or none = 1. (23) Fovea: normal = 0; with spheroid process = 1. (24) Metatarsus I of males: normal = 0; strongly curved = 1. (25) urticating hairs on prolateral palpal femur: absent = 0; present = 1. (26) Urticating setae type VI: absent = 0; present = 1. (27) Coxae: normal = 1; retrolaterally extend = 1.

Bertani 2000, 2001 Characters: (28) Apical keel: absent = 0; small = 1; intermediated = 2; very long = 3. (29) Retrolateral keel: absent = 0; present, not pronounced = 1; present, pronounced = 2. (30) Subapical keel: absent = 0; present, not serrated = 1; present, serrated = 2. (31) Prolateral accesoty keel, under the prolateral inferior keel: absent = 0; present = 1. (32) Prolateral inferior keel: absent = 0; present = 1. (33) Denticulate row in the PIK: absent = 0; present = 1. (34) Prolateral superior keel: absent = 0; presente = 1.

Perafán and Pérez-Miles 2014 Character: (35) Embolus direction: directed ventrolaterally = 0; directed retrolaterally = 1; straight = 2.

(36) Urticating setae type VII: absent = 0; present = 1.

## Results and discussion

### Kankuamo

Taxon classificationAnimaliaAraneaeTheraphosidae

Perafán, Galvis & Pérez-Miles gen. n.

http://zoobank.org/622CB9E5-59D1-4E45-A5CE-21F5248CF9EF

#### Type species.

*Kankuamo marquezi* Perafán, Galvis & Gutiérrez, sp. n.

#### Diagnosis.

**Figure 2.**
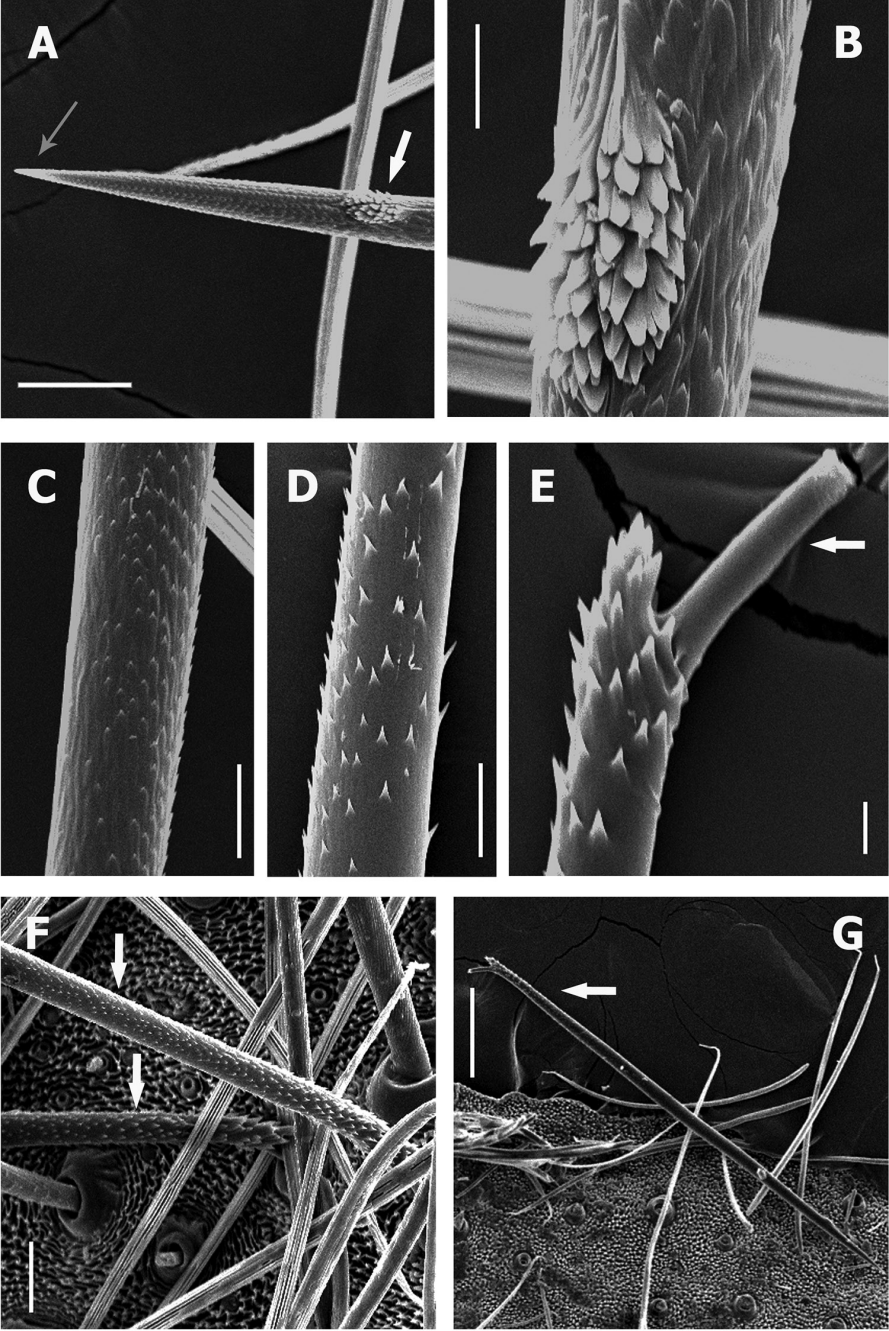
*Kankuamo* gen. n., urticating setae type VII. **A** Distal apex, white arrow indicates patch of lanceolated reversed barbs, grey arrow indicates penetrating tip **B** patch of lanceolated reversed barbs **C** main barbs on distal area **D** main barbs on medial area **E** basal end and detail of main barbs on basal area, white arrow indicates the attachment stalk with the abdomen **F–G** abdomen, dorsal surface, showing setae attachment points, white arrow indicates urticating setae. Scale bars: **A**, **F** = 50 µm; **B**, **E** = 10 µm; **C**, **D** = 20 µm; **E** = 200 µm.

**Figure 3.**
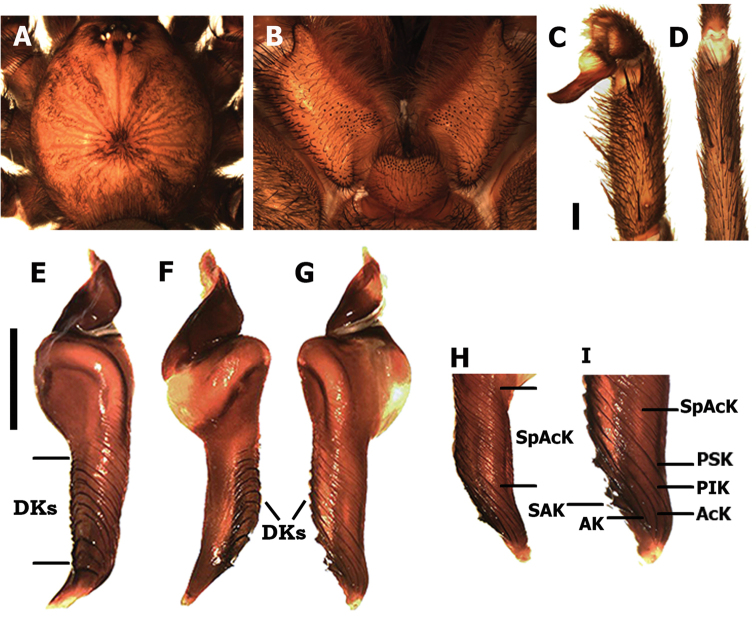
*Kankuamo marquezi* gen. n., sp. n., male. **A** Cephalothorax **B** labium and maxillae **C** right palpal bulb, ventro-prolateral view **D** tibia I in ventral view, showing absence of apophysis **E-I** left palpal bulb: **E** dorsal view **F** retrolateral view **G** prolateral view **H-I** detail of apex. AcK = accessory keels, AK = apical keel, DKs = dorsal keels, PIK = prolateral inferior keel, PSK = prolateral superior keel, SAK = sub-apical keel, SpAcK = supra-accessory keels. Scale bars: **C**, **D** and **E**, **F**, **G** = 1 mm.

Differs from all previously known genera of Theraphosidae by having a distinct type of urticating setae (Fig. 2, see description below), mainly characterized by having a small distal patch of lanceolated barbs arranged in reversed direction, regarding the main barbs, oriented with their tips towards the penetration tip (Fig. 2B). Male differs additionally from other genera by having a curved sub-conical palpal bulb with many conspicuous keels distributed throughout the majority of the subtegulum and embolus, especially developed on the dorsal and prolateral faces, most of them with serrated edges (Fig. 3E–3I). PSK, AcK, PIK, AK and SAK present (*sensu* Bertani 2000), additionally dorsal keels (DKs) and supra-accessory keels (SpAcK). Tibial apophysis on leg I absent (Fig. 3D). Females differ by having spermathecae with a single notched receptacle, with two granulated lobes, and several irregular sclerotized longitudinal striations (Fig. 3D).

#### Etymology.

*Kankuamo* is a noun in apposition and refers to the indigenous people of the Chibcha family from the Caribbean region of Colombia, which inhabits the eastern slope of the Sierra Nevada de Santa Marta, whose language and culture are at endangered. *Kankuamo* gender is neuter.

#### Description.

See description of the type species.

#### Distribution.

Only known from its type locality, Vereda San Lorenzo, Corregimiento Minca, Santa Marta, Magdalena, Colombia, 11.1 N, -74.05 W (Fig. 7).

#### Discussion.

**Figure 4.**
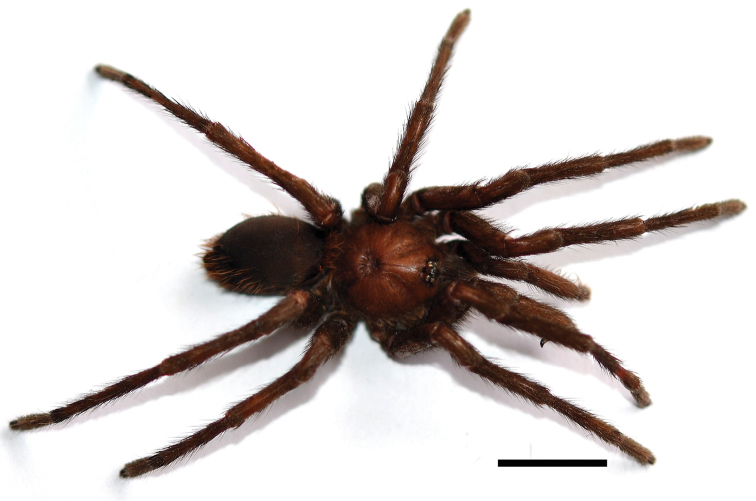
*Kankuamo marquezi* gen. n., sp. n., male, dorsal view of *habitus*. Scale bar = 1 cm.

**Figure 5.**
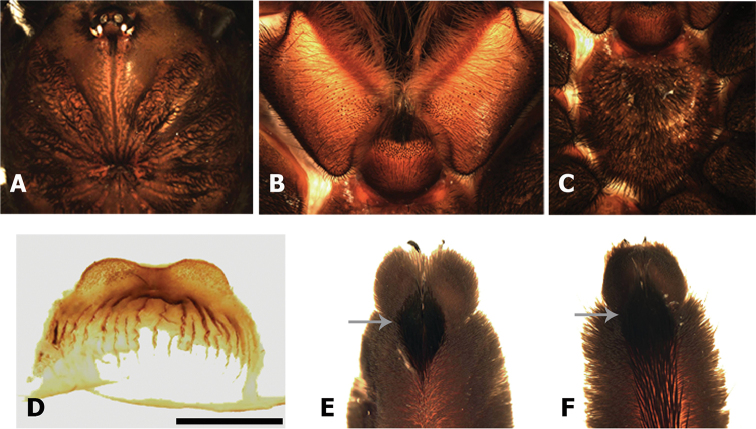
*Kankuamo marquezi* gen. n., sp. n., female. **A** Cephalothorax **B** labium and maxillae ventral **C** sternum **D** spermathecae, dorsal view **E–F** legs tarsi in ventral view **E** tarsus I **F** tarsus IV, arrow indicates distal rhomboidal group of conical setae. Scale bar = 1 mm.

**Figure 6.**
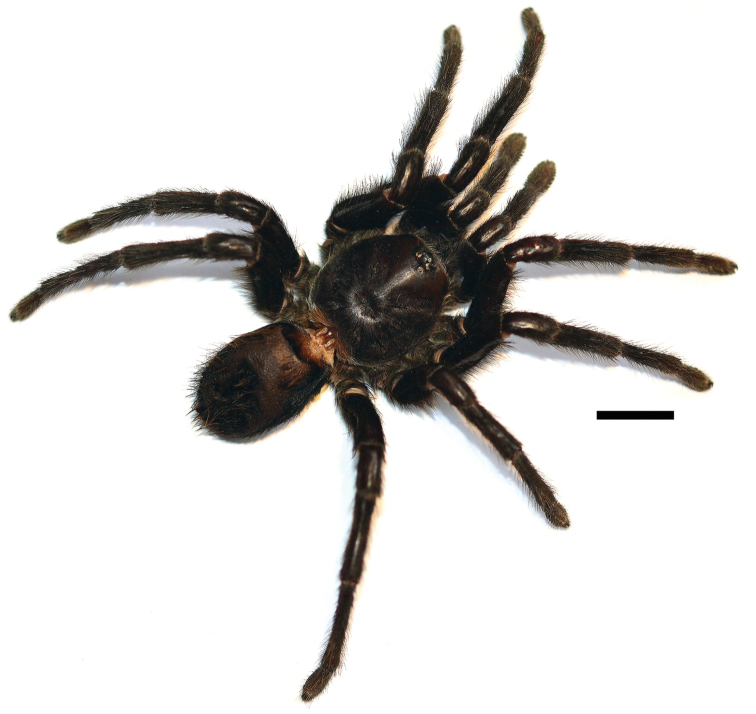
*Kankuamo marquezi* gen. n., sp. n., female, dorsal view of *habitus*. Scale bar = 1 cm.

**Morphology.** *Kankuamo* gen. n. clearly presents the diagnostic characters of the subfamily Theraphosinae (Theraphosidae): male palpal bulb with large and extended subtegulum, and numerous developed keels; legs spinose, with normal scopulae on tarsi and presence of abdominal urticating setae (Figs 2–6). However, this new genus differs dramatically from all previously known genera by their novel urticating setae type and the detailed morphology of male palpal bulb, making it difficult to establish their phylogenetic affinities. The general shape of the palpal bulb resembles approximately those of the genus *Ami* Pérez-Miles, 2008 (mainly *Ami bladesi* Pérez-Miles, Gabriel and Gallon, 2008 and *Ami weinmanni* Pérez-Miles, 2008), but with the subtegulum more elongated in *Kankuamo* gen n. (Fig. 3G). However, the transverse arrangement of bulb keels is also superficially similar to those of some *Acanthoscurria* Ausserer, 1871, or to the ring shaped keel of *Hapalopus* Ausserer, 1875. Numerous distal keels are also known in other genera such as *Lasiodora* C.L. Koch, 1850, *Nhandu* Lucas, 1983 and *Vitalius* Lucas, Silva and Bertani, 1993, but the higher number of keels in *Kankuamo* gen. n. easily distinguish it from all those and other remaining genera in Theraphosinae. Furthermore, the high number of keels make difficult to establish homology with those of the family as Bertani (2000) suggested. Considering the extension and positional similarity we tentatively propose the presence of PSK, AcK, PIK, AK, and SAK, and we also propose the name supra-accessory keels (SpAcK) for those placed on proximal subtegulum (Fig. 3H, 3I) and dorsal keels (DKs) for those placed principally on dorsal face (Fig. 3E–3G). The absence of tibial apophysis is shared with *Agnostopelma* Pérez-Miles and Weinmann, 2010; *Aguapanela* Perafán, Cifuentes and Estrada, 2015; *Metriopelma* Becker, 1878; *Nhandu*; *Sericopelma* Ausserer, 1875 and *Theraphosa* Thorell, 1870.

**Figure 7.**
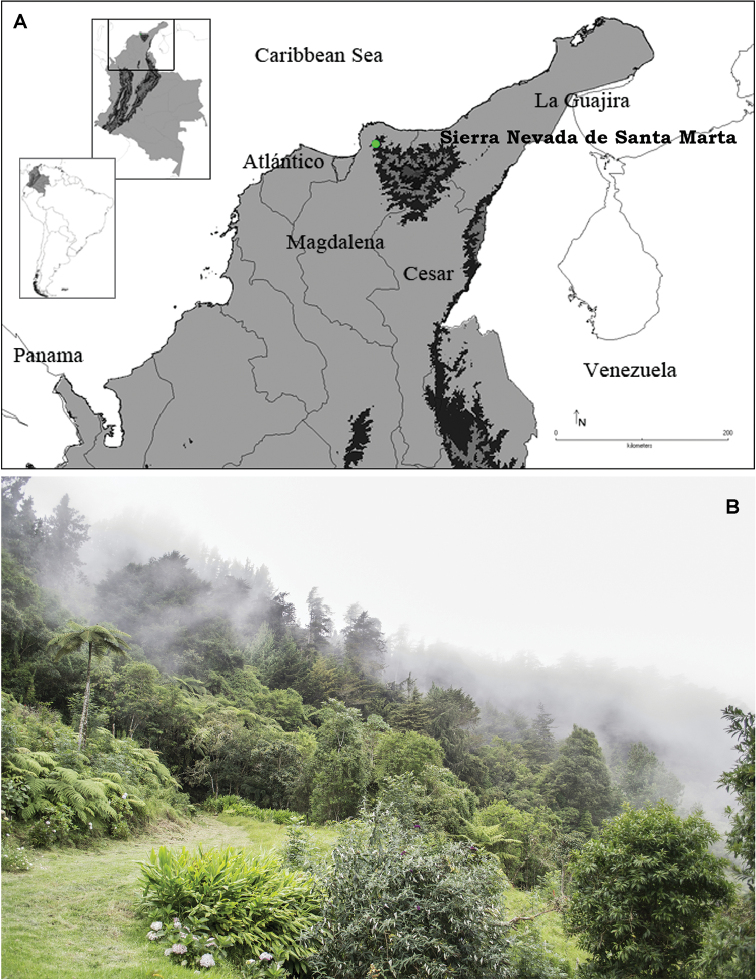
**A** Map of northern Colombia showing the distribution of *Kankuamo marquezi* gen. n., sp. n. **B** habitat of *Kankuamo marquezi*, Cuchilla San Lorenzo, Sierra Nevada de Santa Marta.

The spermathecae of *Kankuamo* gen. n. are similar to those of Theraphosinae genera with only one entire receptacle (e.g. *Brachypelma* Simon, 1891, *Megaphobema* Pocock, 1901, *Mygalarachne* Ausserer 1871, *Sericopelma* Ausserer, 1875, *Theraphosa* Thorell, 1870), but notched and longitudinally striated (Fig. 5D).

Accordingly, *Kankuamo* gen. n. clearly differs from all genera of Theraphosidae known by the urticating setae type and male palpal bulb characters.

#### Cladistic relationship.

A search using equal weights found 171 most parsimonious tress and the strict consensus of these did not provide any resolution. Search with implied weighting and different concavity indices (k = 3 to 12) found between 3 and 10 shortest trees. The strict consensus of each of these tress recovered different topologies, but between k = 8 and k = 12 the topology of strict consensus did not vary, for this reason we have selected this topology to test *Kankuamo* gen. n. affinities (Fig. 1). It is noted that this new phylogeny analysis of Theraphosinae is an incomplete analysis including only half of the Theraphosinae genera and relatively few characters.

*Kankuamo* gen. n. was resolved as the sister group of *Metriopelma*, supported by the character “femur IV without retrolateral scopula”. Both genera also share the lack of the apophysis on males and a spermathecae with seminal receptacles completely fused. *Kankuamo* gen .n. presents a reversion to state (0) on character 16 “absence the urticating setae type I”, a parallelism of the state (1) on character 31 “presence the accessory keels under prolateral inferior keel” and an autapomorphic character “presence of urticating setae type VII”.

*Kankuamo* gen. n. and *Metriopelma* were resolved as sister genera within the largest clade of our selected topology. The relationship of *Kankuamo* gen. n. + *Metriopelma* as the sister group of the clade (*Theraphosa* (*Sericopelma* (*Brachypelma* (*Megaphobema* (*XenesthisPamphobeteus*) (*Sphaerobothria* (*Phormictopus* (*CyrtopholisAcanthoscurria*))))))) is supported by “absence of retrolateral keel” and “absence of sub-apical keel”, with some homoplasies. The *Theraphosa* clade is supported by two synapomorphies “apical region of palpal bulb with cancave-convex aspect” and “apical keel very long”.

### Kankuamo marquezi

Taxon classificationAnimaliaAraneaeTheraphosidae

Perafán, Galvis & Gutiérrez sp. n.

http://zoobank.org/7EF097DE-5147-4995-985C-35504F164C61

Figs 3 , 4 , 5 , 6 and Table 2

#### Type material.

Holotype male from Colombia, Magdalena, Santa Marta, Corregimiento Minca, Sector San Lorenzo, 2200m above sea level, 11.11 N, -74.058 W, 30-Aug-2014, *leg.* W. Galvis and J. Moreno (ICN-Ar 7983). Allotype female, same data as the holotype (ICN-Ar 7983). Paratypes: one female, same data as the holotype (ICN-Ar 7984); one male from the same locality as the holotype, 11.1 N, 74.05 W, 9-10-Sept-2014, *leg.* Miguel Gutierrez (ICN-Ar 7985).

#### Etymology.

The specific epithet is a noun in genitive in honor to Gabriel García Márquez (Aracataca, Colombia, 1927 - Mexico D.F., Mexico, 2014), who was a renowned Colombian writer, considered one of the most significant authors of the 20th century, and awarded the 1982 Nobel Prize in Literature for “One hundred years of solitude”.

#### Diagnosis.

See diagnosis of the genus.

#### Description.

*Male* (holotype ICN-Ar 7982) (Figs 3 and 4). Total length, not including chelicerae or spinnerets 27; including chelicerae 30. Carapace length 12, width 11. Abdomen length 14. PLS with three segments, distal digitiform, basal length 2, medial 1.3, apical 1.6. PMS well developed mono-segmentated, length 1.3. Anterior eye row slightly procurved, posterior slightly recurved. Eyes sizes and interdistances: AME 0.43, ALE 0.56, PME 0.4, PLE 0.43, AME-AME 0.36, AME-ALE 0.13, ALE-ALE 1.26, PME-PME 0.93, PME-PLE 0.06, PLE-PLE 1.46, AME-PME 0.16, ALE-PLE 0.1. OQ elevated sub-rectangular, length 2.1, width 1.33, clypeus 0.33. Fovea transverse deep, straight, width 2.16. Cephalic area slightly raised, thoracic striations slightly conspicuous (Fig. 3A). Basal segments of chelicerae with 9 well-developed teeth on furrow promargin and 19/21 (left/right) small teeth on the proximal area of furrow, intercheliceral tumescense absent. Labium trapezoidal (Fig. 3B), length 1.46, width 2.16, with 56 cuspules. Maxillae sub-rectangular (Fig. 3B), with 56/78 (left/right) cuspules restricted on the proximal prolateral angle. Labio-sternal junction narrow in the middle with two lateral nodules. Sternum length 4.7, width 4.6, with 3 pairs of sigilla; oval, smaller pair anterior, larger pair posterior, anterior pair half distanced at half their diameter from margin, posterior pairs distanced less than 1/3 of their diameter. Superior tarsal claws with teeth on proximal half: I 4 teeth; II-IV 5 teeth. Tarsal scopulae: I-IV scopulated with distal rhomboidal group of conical setae (as Figs 5E and 5F); I and II entire; III and IV divided by a medial stripe of longer conical setae, wider in IV. Metatarsal scopulae extent: I and II scopulate on distal half; III distal 1/4; IV apically, little scopulate. Stridulatory setae absent. Type VII urticating setae present (see description), located on dorsal patch of the abdomen. Metatarsus I straight. Tibia I without apophysis (Fig. 3D). Palpal tibia with spines on ventral and prolateral faces (Fig. 3C). Cymbium bilobed. Palpal bulb sub-conical (Fig. 3E–3I), curved, with a wide membranous area between the subtegulum and tegulum. Distal sclerites of palpal bulb with many conspicuous keels distributed throughout most tegulum and embolus. Eleven large semicircular keels on the dorsal faces (DKs), most of them with serrated edge (Fig. 3E). Presence of PSK, AcK, PIK, AK, SAK, and approximately 14 smaller keels present on the prolateral face of proximal subtegulum (SpAcK) (Fig. 3G–3I). Colour (in alcochol): Cephalothorax and legs light brown with black setae. OQ with black stains and surrounded by black setae, cephalothorax with black stripes. Abdomen brown with golden setae. Iridescent scopulae and claw tuft.

Spination (proximal to distal): Femur: palp: 0V, 0D, 0-0-1P, 0R; I: 0V, 1-1-0D, 0-1-1P, 0-1-2R; II: 0V, 1-1-0D, 1-1-3P, 1-5-2R; III 0V, 1-1-0D, 0-2-1P, 0-2-3R; IV: 0V, 3-2-0D, 1-2-1P, 0-1-2R. Patella: palp: 0V, 0D, 0-2-0P, 0R; I: 0-2-2V, 0D, 0-2-0P, 0R; II: 0-0-2V, 0D, 0-2-0P, 0R; III: 0V, 0D, 0-3-0P, 0-1-0R; IV: 0V, 0D, 0-3-0P, 0-1-0R.Tibia: palp: 2-1-2V, 0D, 2-2-2P, 0R; I: 4-6-2apV, 0D, 0-2-2P, 0-1-1R; II: 3-5-2apV, 0D , 0-1-1P, 0-2-0R; III: 3-3-2apV, 0D, 2-2-1P, 2-2-1R; IV: 3-3-2-1-2apV, 0D, 2-2-2P, 1-2-2-2apR. Metatarsus: I: 2-3-1V, 0D, 0-1-1apP, 0-1-1apR; II: 3-3-1V, 0D, 0-1-1-1apP, 0-1-1apR; III: 4-4-2-1apV, 0-0-2D, 2-2-1-1apP, 1-2-1-1apR; IV: 5-5-3-1apV, 0-0-2D, 2-3-1-1apP, 2-2-1-1apR. Tarsus: palp and legs: 0. Legs and palpal segments lengths in Table 1.

Female (allotype ICN-Ar 7983) (Figs 5 and 6). Total length, not including chelicerae or spinnerets 39; including chelicerae 44.5. Carapace length 17.1, width 16.3. Abdomen length 17.4. PLS with three segments, distal digitiform, basal length 2.25, medial 1.25, apical 2.45. PMS well developed mono-segmentated, length 1.9. Anterior eye row slightly procurved, posterior recurved. Eyes sizes and interdistances: AME 0.5, ALE 0.5, PME 0.53, PLE 0.66, AME-AME 0.5, AME-ALE 0.33, ALE-ALE 2, PME-PME 1.3, PME-PLE 0.06, PLE-PLE 1.83, AME-PME 0.16, ALE-PLE 0.33. OQ elevated sub-rectangular, length 1.93, width 2.83, clypeus 0.65. Fovea transverse deep, straight, width 3. Cephalic area slightly raised, thoracic striations conspicuous with black setae (Fig. 5A). Basal segments of chelicerae with 10 well-developed teeth on furrow promargin and 21/17 (left/right) small teeth on the proximal area of furrow, intercheliceral tumescense absent. Labium trapezoidal (Fig. 5B), length 2.27, width 3.06, with 99 cuspules. Maxillae sub-rectangular (Fig. 5B), with 159/164 (left/right) cuspules restricted on the proximal prolateral angle. Labio-sternal junction narrow in the middle with two lateral nodules. Sternum (Fig. 5C) length 7.4, width 7, with three pairs of oval sigilla; posterior sigilla the largest, all of them separated from the margin by approximately their diameter. Superior tarsal claws with teeth on proximal half; palp and leg I, three teeth, smaller in palp; II four teeth; III-IV five teeth. Tarsal scopulae: palp and I-IV scopulated with distal rhomboidal group of conical setae (Fig. 5E–5F); palp and legs I-II entire; III and IV divided by a medial stripe of longer conical setae, wider in IV. Metatarsal scopulae extent: I scopulate on distal 2/3; II distal half; III distal 1/3; IV scopula absent. Stridulatory setae absent. Type VII urticating setae present (see description), located on dorsal patch of the abdomen. Spermathecae with two short wide rounded and granulated seminal receptacles, fused widely on a sub-rectangular wide basal plate, with several irregular sclerotized striations perpendicular to the basal edge (Fig. 5D). Color (in alcohol): darker than male. Iridescent scopulae and claw tuft.

Spination (proximal to distal): Femur: palp: 0V, 0-1-0D, 0-0-3P, 0-0-1R; I: 0V, 0-1-0D, 0-0-3P, 0R; II: 0V, 0-2-0D, 0-0-3P, 0-1-0R; III: 0V, 0D, 0-2-1P, 1-3-1R; IV: 0V, 1-0-0D, 0-0-3P, 0-0-1R. Patella: palp: 0V, 0D, 0-2-0P, 0R; I: 0V, 0D, 0-1-0P, 0R; II: 0V, 0D, 0-2-0P, 0R; III: 0V, 0D, 0-2-0P, 0R; IV: 0V, 0D, 0-1-0P, 0R. Tibia: palp: 1-4-4V, 0D, 0-3-0P, 0-1-0R; I: 0-1-2V, 0D, 1-1-0P, 0R; II: 0-2-2V, 0D, 0-2-0P, 0R; III: 3-3-3V, 0D, 2-1-2P, 1-2-1R; IV: 2-2-2V, 0D, 2-2-1P, 2-2-1R. Metatarsus: I: 1-5-1V, 0D, 0-1-1P, 0R; II: 1-5-1V, 0D, 0-0-1P, 0R; III: 4-3-5V, 0-0-2D, 2-2-2P, 1-3-1R; IV: 5-4-5V, 0-0-2D, 3-3-2P, 1-2-2R. Legs and palpal segments lengths in Table 1.

**Table 2.** Length of legs and palp segments in millimeters of holotype male/allotype female *Kankuamo marquezi* gen. n., sp. n.

Segments	Palp	I	II	III	IV
**Femur**	6.2 / 8.0	9.0 / 10.8	8.5 / 10.2	8.4 / 9.7	9.5 / 11.5
**Patella**	2.8 / 4.5	4.5 / 6.0	3.9 / 5.7	3.5 / 5.0	3.8 / 5.4
**Tibia**	5.8 / 6.0	7.4 / 8.0	6.9 / 7.6	6.3 / 7.0	8.0 / 9.3
**Metatarsus**	-	7.0 / 8.0	7.0 / 7.5	7.8 / 8.6	10.9 / 12.0
**Tarsus**	2.1 / 5.4	4.3 / 4.5	4.5 / 4.5	4.2 / 4.4	4.5 / 4.5
**Total**	16.9 / 23.9	32.2 / 37.3	30.8 / 35.5	30.2 / 34.7	36.7 / 42.7

#### Distribution.

See distribution of the genus (Fig. 7).

#### Natural history.

*Kankuamo marquezi* sp. n. inhabits Cuchilla San Lorenzo from Sierra Nevada de Santa Marta National Natural Park. Cuchilla San Lorenzo is located in its northwestern flank, in a gradient of altitude from 2000–2300 meters above sea level, in life zone of lower montane wet forest (Espinal and Montenegro 1963). The Sierra Nevada de Santa Marta is an isolated mountain range separated from the Andes chain. The locations where the specimens were collected are covered mainly by shrubby plants of the families Arecaceae and Chrysobalanaceae (Cuadrado-Peña 2005), and inhabited by the snakes genus *Atractus* and frogs of the genera *Atelopus*, and the endemic species from Sierra Nevada de Santa Marta *Ikakogi tayrona* and *Geobatrachus walkeri* (MG personal observations).

#### Description of urticating setae type VII.

##### (Fig. 2)

**Morphology.** Urticating setae differ from body covering setae by the insertion feature through a stalk (types I, II, III and IV) or attached into a specialized socket (types V and VI) that facilitates detachment, plus presence of a penetrating acute tip, and barbs or scales that aid embedding them into targets (Cooke et al. 1972, Bertani and Guadanucci 2013).

Urticating setae type VII are located in a dorsal wide area of the abdomen intermixed with the covering setae, and attached to the cuticle by a thinner stalk, to facilitate their release (Fig. 2G). Setae length is 1122±40 µm, and width is 33±3 µm, length/width ratio 34 (n=10), with a very sharp penetrating tip on the distal apex, opposite to the stalk (Fig. 2A). The stalk of the setae is larger than in the other urticating types (Fig. 2E), approximately length 45±3 µm, and width 10±1 µm. The main shaft is straight, having small barbs (main barbs) that extend along the whole setae (Fig. 2C, D), plus a small oval patch of lanceolated reversed barbs near the penetrating tip (Fig. 2A, B).

Main barbs are subtriangular denticles not homogeneous in size and density, longer on the basal region (Fig. 2E) and densest on the distal (Fig. 2A), and oriented with the acute extreme toward the basis of the setae. Main barbs length on distal area less than 1 µm (Fig. 2A, C), medial area around 3 µm (Fig. 2D), and on basal area of 9±2 µm (Fig. 2E).

The patch of lanceolate barbs its located sub-apically at a distance approximately of 160µm from the tip to the patch centre (Fig. 2A, 2B). Patch approximately 30 µm length and width 15 µm, with around 50 lanceolate barbs. These are arranged in reversed direction, regarding the main barbs (*sensu* Cooke et al. 1972), oriented with the acute extreme toward the penetration tip. They are longer, broader and less acute than the main barbs (Fig. 2B); with each lanceolate barb of length 5±1 µm and width 2.5±0.5 µm.

**Discussion.** The newly characterized type VII urticating setae resemble the type II found in Aviculariinae (Cooke et al. 1972, Bertani and Marques 1996, Bertani and Guadanucci 2013), but the main differences from those are: 1) The presence of a sub-apical oval patch of lanceolated reversed barbs, 2) The penetration tip is apical (proximal in type II), 3) The main barbs are oriented towards the base of the setae (oriented towards the apex on type II), 4) The proximal end of the setae is covered by larger main barbs, and 5) Usually, the stalk remains attached to the seta (the stalk remains attached to the body in type II).

The similarities of the morphology and size of setae type VII with type II Aviculariinae (see Cooke et al. 1972, figs 11–12, 20–21) suggest a releasing mechanism by direct contact, as indicated for *Avicularia* (excepting *Avicularia versicolor*, see Bertani et al. 2003), *Iridopelma*, *Pachistopelma* and *Typhochlaena* (Bertani 2012). These tarantulas direct the abdomen toward the stimulus and transfer the urticating setae when the abdomen of the spider touches against the target (Bertani and Marques 1996). Contrary, in Theraphosinae, the various types of urticating setae are dislodged by friction of the hind legs against the dorsum of the abdomen, and air-transported (Cooke et al. 1972).

Bertani and Marques (1996) proposed that the differences of the shape, size and thickness between Theraphosinae and Aviculariinae urticating setae could explain the two releasing mechanisms. The morphological characteristics of all then known urticating hair types in Theraphosinae, particularly their light weight and aerodynamics, would allow them to float through the air. They suggest that short and/or thin setae with a ratio of 100:1 or 200:1 are able to float through the air. Bertani et al. (2003) compared length/width ratio between urticating setae type II and III and concluded that all airborne setae (including *Avicularia versicolor* type II) are narrow, mean width ranging from 6 to 7 µm, and length/width ratio ranging from 98.3 to 208.3. In contrast, for urticating setae released by direct contact with the target, the width was greater, ranging from 15 to 22 µm and the length/width ratio of 37.3-46.8 (see Bertani et al. 2003, Table 1). Here, the newly described urticating setae type VII width is 33±3 µm and length/width ratio 34, which therefore represents the thickest setae known until now, and with the lowest known length/width ratio, aspects that together reinforce the hypothesis that their releasing mechanism is by direct contact.

Furthermore, the larger size, broader shape and often dispersed arrangement of many of the main barbs of Theraphosinae urticating setae (types I, III, VI, VI) involved in their urticating effects, presumably often contribute their ability to float in the air. Conversely, in both urticating setae types II and VII, which presumably do not float by air, the main barbs are only residual denticles, being much smaller than those of other types with known air dispersal.

The differences in the position of the penetrating tip between type VII and II also suggest a different penetration mechanism. On first contact with the target, the urticating setae type II pivots on its stalk so the apical end moves away from the target, while the basal penetration tip (which is actually adjacent to the stalk) instead lodges into the target as the stalk releases (see Bertani and Marques 1996, fig. 3). By contrast, the apical penetrating tip of the urticating setae type VII (at the opposite end to the stalk) is able to penetrate directly into the target, at the first contact with the object.

**Figure 8.**
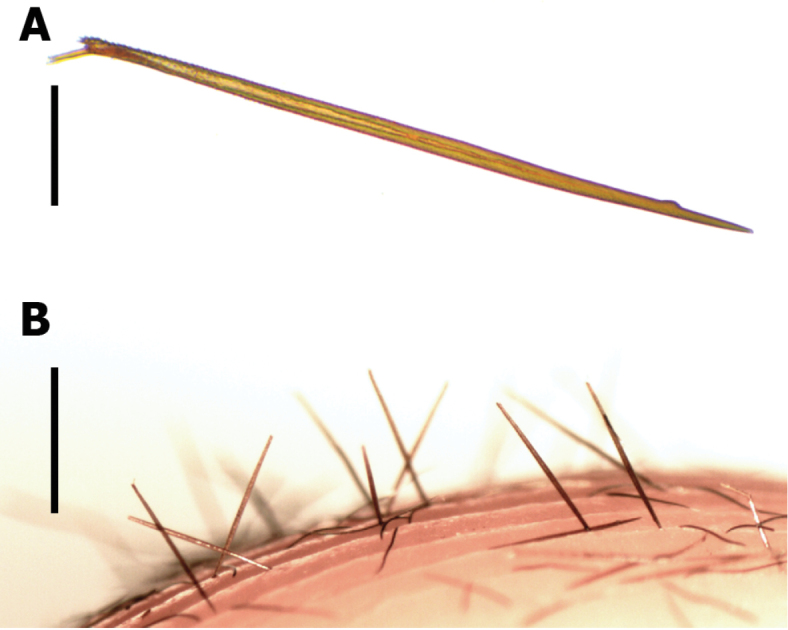
Urticating setae type VII **A** setae viewed in optical microscope **B** urticating setae embedded into the finger skin. Scale bars: **A** = 200 µm; **B** = 1 mm.

Experimentally, the possible mechanism of action of the urticating setae type VII was observed while handling specimens in alcohol. These urticating setae easily perforated the skin of human fingers perpendicularly. The dorsum of the tarantula’s abdomen was touched intentionally, and on further examination of the affected fingers with a stereoscope microscope, many of these setae were found embedded in the skin (Fig. 8). These setae were firmly fixed into the skin but none penetrated more than a third of their length. When we attempted to remove them from the skin, these setae were easily broken at their distal end. Based on our observations together, our assumption is that the patch of the lanceolate reverse barbs in the distal area can serve as a breakpoint into the skin, ensuring that the apex of the seta remains within the tissues.

Another aspect to consider is that all previously known species with contact urticating setae have leg spines absent or reduced (Cooke et al. 1972, Bertani et al. 2003). *Kankuamo* gen. n. therefore represents a remarkable exception to this suggested character association because, in contrast, they do have legs with some spines, mainly conspicuous on metatarsi and tarsi of hind-leg pair IV. Leg spines in other theraphosids have been functionally associated with releasing of airborne urticating setae by facilitating rubbing, with the exception of *Avicularia versicolor* which use claw tufts for rubbing (Cooke et al. 1972, Bertani and Marques 1996, Bertani and Guadanucci 2013). One possible interpretation is the plesiomorphic retention of contact setae could reflect a close phylogenetic relationship between Aviculariinae and Theraphosinae, as proposed by Pérez-Miles et al. (1996). In this scenario, the additional development of posterior leg spines (such as found in *Kankuamo* gen. n.) could have facilitated the evolutionary transformation of contact setae into derived airborne setae, which are now widespread among Theraphosinae. However, if contact setae are interpreted as derived, the posterior leg spines present in *Kankuamo* gen n. could be regarded as a plesiomorphic retention.

## Conclusions

*Kankuamo* gen. n. fits the diagnostic characters of Theraphosinae, but also shows a very divergent palpal bulb morphology and the presence of a new abdominal urticating setae type. These setae are unique, and here are proposed to be the only contact released urticating setae yet known within the Theraphosinae, although this release mechanism was previously well known only for Aviculariinae. Also, the supernumerary keels on the male palpal bulb clearly distinguish it from all known theraphosid species. *Kankuamo* gen. n. was resolved as the sister group of *Metriopelma* on our preferred phylogeny of Theraphosinae.

## Supplementary Material

XML Treatment for Kankuamo

XML Treatment for Kankuamo marquezi
